# Chloride ions in health and disease

**DOI:** 10.1042/BSR20240029

**Published:** 2024-04-29

**Authors:** Satish K. Raut, Kulwinder Singh, Shridhar Sanghvi, Veronica Loyo-Celis, Liyah Varghese, Ekam R. Singh, Shubha Gururaja Rao, Harpreet Singh

**Affiliations:** 1 Department of Physiology and Cell Biology, College of Medicine, The Ohio State University, Columbus, OH, U.S.A.; 2 Department of Molecular Cellular and Developmental Biology, The Ohio State University, Columbus, OH, U.S.A.; 3 Raabe College of Pharmacy, Ohio Northern University, Ada, OH, U.S.A.

**Keywords:** Anion transport, Chloride channels, Chloride ions, Chloride transporters, Ion homeostasis

## Abstract

Chloride is a key anion involved in cellular physiology by regulating its homeostasis and rheostatic processes. Changes in cellular Cl^−^ concentration result in differential regulation of cellular functions such as transcription and translation, post-translation modifications, cell cycle and proliferation, cell volume, and pH levels. In intracellular compartments, Cl^−^ modulates the function of lysosomes, mitochondria, endosomes, phagosomes, the nucleus, and the endoplasmic reticulum. In extracellular fluid (ECF), Cl^−^ is present in blood/plasma and interstitial fluid compartments. A reduction in Cl^−^ levels in ECF can result in cell volume contraction. Cl^−^ is the key physiological anion and is a principal compensatory ion for the movement of the major cations such as Na^+^, K^+^, and Ca^2+^. Over the past 25 years, we have increased our understanding of cellular signaling mediated by Cl^−^, which has helped in understanding the molecular and metabolic changes observed in pathologies with altered Cl^−^ levels. Here, we review the concentration of Cl^−^ in various organs and cellular compartments, ion channels responsible for its transportation, and recent information on its physiological roles.

## Introduction

Chloride (Cl^−^) is the most abundant ion in humans after sodium [1] and accounts for 70% of the total anions in extracellular fluid (ECF) [2]. There are approximately 115 g of Cl^−^ in an average human adult body, making up to 0.15% of the total body weight as a key macromineral [3]. Cl^−^ are vital for maintaining osmotic pressure, muscle movement, and acid-base balance in the body [3]. Cl^−^ homeostasis is generally overlooked but is known to govern several key physiological functions inside and outside the cell [2,4–9]. Along with cations, Cl^−^ is responsible for maintaining ionic homeostasis, osmotic pressure, and acid–base balance. Therefore, disturbances of Cl^−^ levels are indicative of metabolic disorders including hypochloremic metabolic alkalosis and hyperchloremic metabolic acidosis [2,10]. Cl^−^ does not follow the electrochemical equilibrium in most mammalian cells. In several cells, including primary sensory neurons, leukocytes, epithelial, sympathetic ganglion, and muscle cells, intracellular Cl^−^ is maintained above equilibrium levels. The transport of Cl^−^ occurs via channels, exchangers, and co-transporters that utilize chemical as well as electrical gradients [2,11].

Cl^−^ is a component of a daily diet in the form of sodium chloride (NaCl). It is classified as an electrolyte as it carries a negative charge along with its positive counterparts, K^+^ and Na^+^. Cl^−^ is mainly found in a diet consisting of seaweed, rye, vegetables such as lettuce, tomatoes, olives, celery, fruits such as apples, melons, berries, and bananas, as well as red meats [12–14]. Most of the Cl^−^ also comes from added salt in several food preparations [14]. The dietary intake levels for Cl^−^ vary with development as shown in Table 1: 0.3 g/day for infants aged 7–11 months, 1.7 g/day for children aged 1–3 years, 2.0 g/day for children aged 4–6 years, 2.6 g/day for children aged 7–10 years, 3.1 g/day for children aged 11–17 years, and 3.1 g/day for adults, including pregnant and lactating women [8]. Cl^−^ deficiency is extremely rare as the average diet is high in NaCl [8]. A loss of Cl^−^ is accompanied by a loss of sodium (Na) ions, observed in patients with prolonged diarrhea, vomiting, or excessive sweating [15,16]. Additionally, diuretics or high blood glucose levels can result in decreased Cl^−^ levels [17]. In contrast, hyperchloremia (above the reference range of 97−107 mmol/L) is caused by an excessive intake of NaCl, severe dehydration, or metabolic abnormalities [3]. Excreted Cl^−^ levels in urine are independent of Cl^−^ intake, making it difficult to evaluate the status of Cl^−^ in the body [17]. There are limited studies where the role of Cl^−^ was evaluated in pathological conditions [2]. Only studies on cardiovascular diseases tend to incorporate a control such as normal Na^+^ and low Cl^−^ levels to implicate Cl^−^ in determining the outcome and survivability of patients [5,7,18–23].

**Table 1:** Chloride levels in various human organs

Organ	Fetus (mM)	Infant (mM)	Adult (mM)
Skin	90–96	67–72	71
Heart	41	45–50	45
Liver	57–62	42–55	38
Kidney	60–67	61	58
Brain	72	66	41
Blood	96–106	90–110	98–106

In various human organs, Cl^−^ levels (mM/L) decrease with age, except for the heart and blood. In the heart and blood, Cl^−^ levels show a small increase. All values were obtained from previous studies [26,82].

Cl^−^ is specifically necessary for the formation of hydrochloric acid (HCl) in the stomach, which activates several gastric enzymes involved in the digestion [24]. The concentration of Cl^−^ in the stomach is 150 mM, whereas in the blood it is 98–106 mM [25]. Therefore, Cl^−^ must be secreted in the lumen against the concentration gradient. The membrane potential at the apical surface of the resting cell is −70 mV [24]. This facilitates Cl^−^ secretion against the electrical gradient. In conditions like excessive vomiting, the loss of stomach content results in an abnormal feedback mechanism for acid-mediated secretion of digestive enzymes [24]. Several clinical conditions are related to the decreased concentration of Cl^−^ in the serum, termed hypochloremia (typically below the reference range of 97−106 mmol/L)-, which manifests in metabolic alkalosis [26]. Conversely, high Cl^−^ concentration above the reference range results in hyperchloremia. An excessive loss of bicarbonate tends to cause a proportional increase of Cl^−^ [27] as a result of excessive carbonate loss observed during severe diarrhea [2,26] or the intake of certain medications such as acetazolamide and triamterene.

Cl^−^ is a key ion of the extracellular fluid compartment (ECF), and with a concentration of 155 mM, it makes up 66% of all the ECF anions [27]. In addition to ECF, Cl^−^ is also present in the intracellular spaces, albeit at lower concentrations [27]. The slight concentration difference between two different compartments is due to capillary impermeability to proteins such as albumin [27]. The intracellular Cl^−^ concentration depends on the cell types and function with respect to other ions [4]. On average, the intracellular concentration of Cl^−^ ranges from 5 to 60 mM [28]. Muscle cells have a resting potential of approximately −70 mV and a low Cl^−^ concentration of 3–4 mM [29]. However, cells with high membrane potential, such as erythrocytes, have a higher concentration of Cl^−^ of around 70 mM [30]. This higher concentration is essential in moving Cl^−^ into and out of the cell effectively during the phenomenon of *‘chloride shift*’ between the plasma and the red blood cells [30,31].

**Figure 1:**
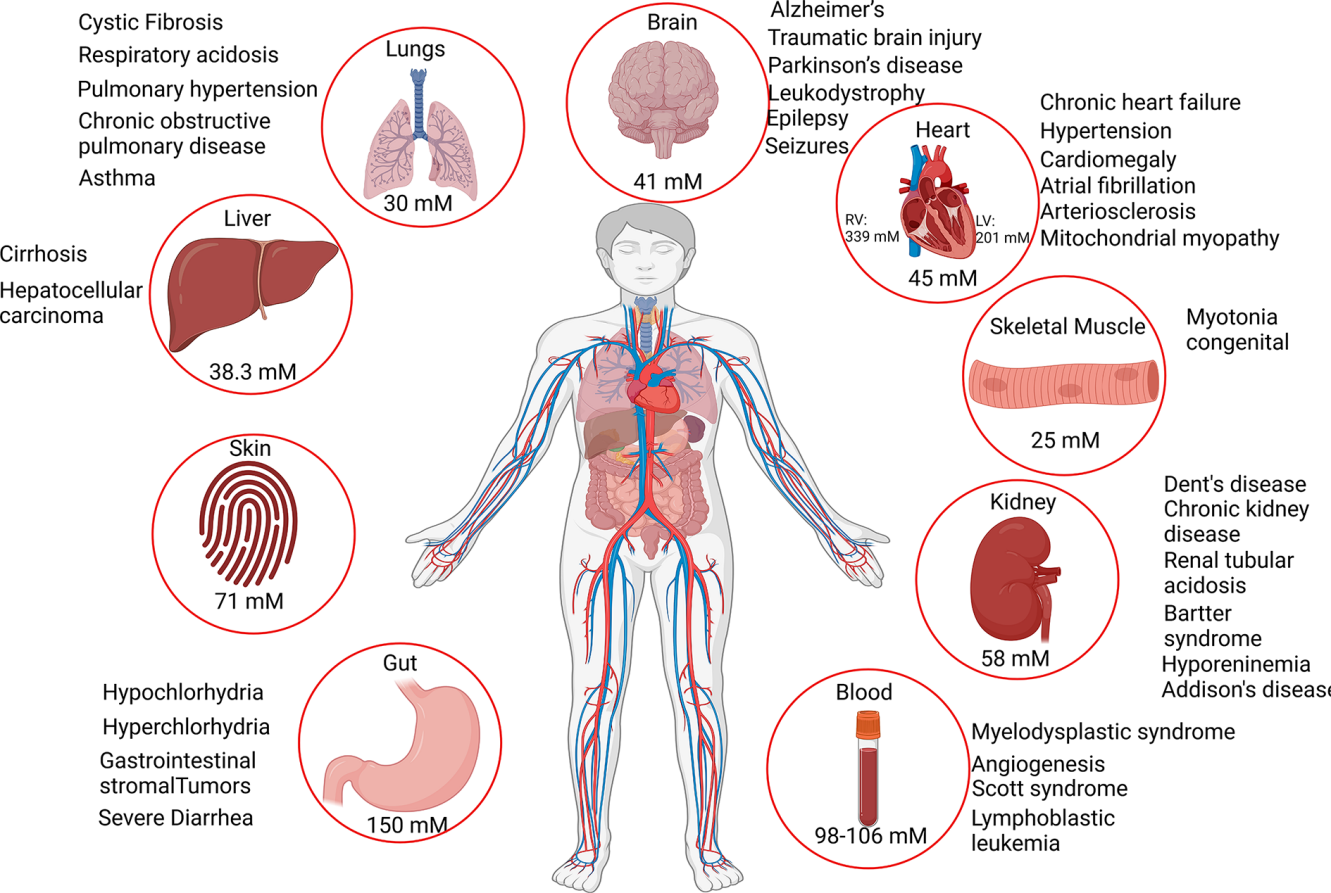
Chloride concentrations in adult human organs The Cl^−^ concentration in adult human organs varies in different organ systems. The Cl^−^ concentration in the brain (41 mM), heart (overall 45 mM, and specifically right ventricle 339 mM and left ventricle 201 mM), muscle (25 mM), kidney (58 mM), blood (98–106 mM), gut (150 mM), skin (71 mM), liver (38 mM), and lungs (30 mM). Associated human diseases for various organs are highlighted. All the values were obtained from previous studies [26–28,31,36,40,50,81–83,88,116,156,157,169,195–206]. Images were generated by Biorender.

**Figure 2:**
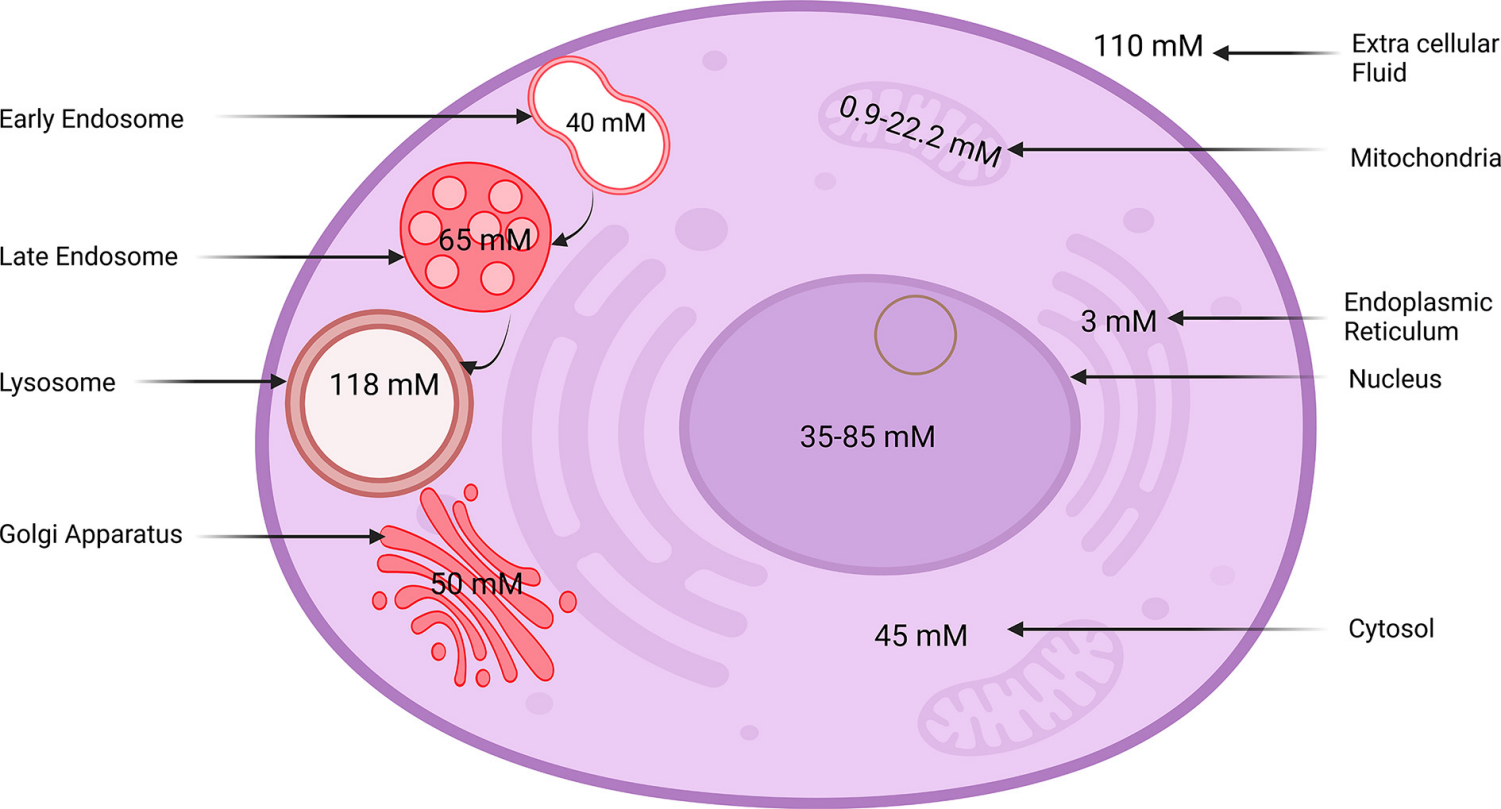
Schematic representation of chloride concentration in the cell organelles The Cl^−^ concentration in an extracellular and cellular compartment maintains cellular homeostasis. The extracellular Cl^−^ concentration (110 mM), cytosol (36 mM), early endosome (40 mM), late endosome (65 mM), lysosome (118 mM), mitochondria (0.9–22.2 mM), nucleus (35–85 mM), endoplasmic reticulum (3 mM), and Golgi apparatus (50 mM) [83,133,134,169,175,192,207,208].

In this review, we summarize the recent information on the role of Cl^−^ in organ (Figure 1) and cellular (Figure 2) physiology. Although abnormal Cl^−^ levels are indicators of several physiological conditions, the ion channels and transporters that conduct ions remain understudied compared with their cationic counterparts [32].

### Chloride and organ systems

Cl^−^ levels in the body are regulated by kidneys [33]. In the glomerular ultrafiltrate, Cl^−^ is the most prevalent ion after sodium. Most of the Cl^−^ is filtered and reabsorbed in the renal tubules by both active and passive transportation mechanisms [34]. In addition to the kidneys, the intestines also absorb Cl [35]. In this section, we will discuss the role of each organ system and its Cl^−^ levels. During early development and preterm infancy, Cl^−^ levels (Table 1) are influenced by sodium, Cl^−^ intake, and gestational age [36].

### Chloride in the kidneys

The excretion of Cl^−^ is mainly done via the kidneys (Figure 1). Approximately 99% of the Cl^−^ filtered through the kidney gets reabsorbed along with Na^+^ [37]. Therefore, only a small fraction gets excreted [27]. Reabsorption occurs either at the paracellular proximal tubule via Cl^−^ channels and transporters, or at the apical membrane via Cl^−^/anion exchangers or basolateral via Cl^−^/carbonate exchanger [38]. In the kidneys, the proximal tubule and the ascending loop of Henle are responsible for reabsorbing the majority of the filtered Cl^−^ in the body [33]. In contrast, the distal tubule and collecting duct absorb a very small amount of Cl^−^ [39]. However, they still play a significant physiological role in maintaining Cl^−^ homeostasis [40]. Proximal convoluted tubule (PCT) absorbs most of the water and 50% of Cl^−^ along with Ca^2+^, Mg^2+^, and HPO_4_^2−^. In basal membranes, the Na^+^/K^+^ ATPase generates an electrochemical gradient that facilitates the reabsorption of Cl^−^ by Na^+^/ Cl^−^ symporters in the apical membrane. While Na^+^ is actively transported from the basal side of the cell into the interstitial fluid, Cl^−^ and Na^+^ are pumped into the interstitial fluid by a paracellular route between cells through leaky tight junctions.

In the collecting ducts of the kidneys, vacuolar H^+^-ATPase and Slc26a11 regulate pH and renal acid–base secretion [41]. Bicarbonate transporters also cause an uptake of NaCl [42]. All the bicarbonate transporters carry HCO_3_^−^ and/or CO_3_^−^ along with at least one either Na^2+^ or Cl^−^ [42]. In the connecting segments and the collecting tubules of the kidneys, aldosterone, a major mineralocorticoid steroid hormone secreted by glomerulosa cells in the adrenal cortex, is another vital component in facilitating the reabsorption of NaCl [43]. Therefore, a deficiency in this hormone would result in hyperkalemic and hyperchloremic acidosis (Figure 1). The key mechanism involves aldosterone by increasing the number of Na and Cl^−^ transporters in the luminal membrane [44,45]. When tubular reabsorption of Cl^−^ is enhanced, it leads to a Na imbalance and extracellular volume expansion, which causes hypertension and hyporeninemia [5]. Kidneys must adapt to metabolic acidosis and acid-base disturbances [46]. Kidneys mainly adapt to these imbalances via Cl^−^ excretion [47]. Kidneys increase acid secretion by enhancing NH_4_Cl secretion via the apical sodium/hydrogen exchanger (NHE3), which also works in tandem with the Na^+^/K^+^/2Cl^−^ cotransporter [48,49]. When there is a prolonged period without sodium excretion, the lack of ion exchange pushes the system to reabsorb bicarbonate and return pH levels to normal [50]. Recently, the outcome of hypochloremeia was evaluated in patients with decompensated cirrhosis. Surprisingly, hypochloremia increases mortality in patient [51].

### Chloride in the gut

Cl^−^ in the gut comes from the consumption of table salt as well as foods containing other types of Cl^−^ salts. Most of the Cl^−^ is absorbed from the intestines during digestion [52] (Figure 1). Cl^−^ in the intestinal lumen gets absorbed by three different mechanisms: a passive or paracellular pathway, an electroneutral pathway involving the Na/H and Cl^−^/carbonate exchange, and a carbonate-dependent Cl^−^ absorption pathway [35].

Hydrochloric acid in gastric juice is composed of Cl^−^ that is secreted into the stomach [53]. Parietal cells located in the middle part of the glands of the fundus-body region of the stomach produce HCl by secreting H^+^ and Cl^−^ [54]. Hydrochloric acid activates digestive enzymes, controls foodborne microorganisms, limits microorganism growth in the intestine, and facilitates the absorption of several nutrients [53]. At pH below 4.0, gastric juices have an anti-microbial effect [53], which is recognized as a ‘gastric bactericidal barrier’ since 1925 [55]. The H^+^K^+^ATPase (the proton pump) in the basolateral and apical membranes of the gut control the secretion of hydrochloric acid into the stomach [56,57]. Moreover, recently identified Cl^−^ channels can also facilitate the secretion of Cl^−^. Some of these are calcium-activated Cl^−^ channels (CaCC), cystic fibrosis conductance regulator (CFTR), and chloride type-2 (ClC-2) channels [58,59]. Na^+^/K^+^ ATPase pumps, potassium channels, and Na^+^/K^+^/Cl^−^ transporters move Cl^−^ across basolateral membranes [58–60]. Another major function of Cl^−^ in the gut is facilitating water absorption [59]. Cl^−^ contributes to the osmotic gradient needed to regulate water secretion into the gut [61] (Figure 1). As water cannot be actively secreted, the driving force is the osmotic gradient generated by negative ions like Cl^−^, as well as carbonate [58]. Na^+^ participates as the counter ion in the paracellular regions [62].

### Chloride in the brain

Cl^−^ in the brain is associated with the regulation of ionic homeostasis and water concentrations [63]. Water accounts for 80% of the total brain, but its transport needs an osmotic gradient by anions [64]. The balance between transporters and Cl^−^ channels in the plasma membrane regulates and maintains the intracellular concentration of Cl^−^ [65]. Neurons and astrocytes express a plenteous set of Cl^−^ channels and transporters belonging to several protein families with unique modes of regulation and activation [65]. Abnormal levels of Cl^−^ are associated with brain disorders, trauma, hypoxic-ischemic encephalopathy, edema, and post-traumatic seizures (Figure 1) [32]. In the brain, the concentration of Cl^−^ levels is low (Figure 1), but in cerebral spinal fluid, the concentration is around 120 mM [66]. There is mounting evidence that disorders of the nervous system are caused by abnormal homeostasis of the intracellular concentration of Cl^−^ [65]. This also causes significant abnormalities in neuronal excitability and neurotransmission.

In the central nervous system, Cl^−^ channels and transporters (Table 2) are essential for the growth and development of neurons, the uptake of neurotransmitters, intracellular pH regulation, cell volume regulation, control of membrane potential, cell proliferation, apoptosis, and, most importantly, the adjustment of [Cl^−^]i to its equilibrium potential [67]. In neurons and astrocytes, Cl^−^ channels, such as CLIC1 [68–70], are pivotal in regulating ion and water homeostasis as they play a key role in action potential generation and impulse conduction [70]. By regulating the postsynaptic reactions of GABA and glycine neurotransmitters, Cl^−^ plays a critical role in modulating neuronal excitability [71,72]. GABA and glycine receptors are ligand-gated Cl^−^ channels that respond to GABA and glycine neurotransmitters, respectively. When these receptors are activated, they cause an influx or efflux of Cl^−^, depending on the electrochemical potential of Cl^−^ for the cell. These Cl^−^ fluxes lead to inhibitory and sometimes excitatory responses [72]. GABAergic signals are the primary inhibitory transmitters in the adult brain and are an important part of coordinating the assembly of neuronal circuits in the developing brain [73]. GABA is the primary neurotransmitter active within the developing brain and facilitates the proliferation of neuronal progenitor cells [74]. The dysregulation of GABAergic signaling has been linked to a variety of neurological and neurodevelopmental disorders, including epilepsy, schizophrenia, Down’s syndrome (DS), and autism spectrum disorders [75]. In relapsing remitting multiple sclerosis, elevated Cl^−^ levels of ≥123.2 mmol/L were associated with an increased frequency of relapse as compared with patients with a cerebrospinal fluid Cl^−^ level of <123.2 mmol/L [76]. Cl^−^ in cerebrospinal fluid is a key electrolyte in maintaining the ionic homeostasis of the brain and spinal cord [76]. In fact, for a long period, spinal fluid Cl^−^ levels were associated with tuberculous meningitis [77]. Any variability in Cl^−^ concentration in cerebrospinal fluid could result in neurological conditions such as hydrocephalus, meningitis, and encephalitis.

**Table 2:** Chloride ion channels and transporters

Name	Localization	Pathophysiology	Conductance (pS)	Permeability
ClC1	Plasma membrane	Myotonia congenital	1–2	Cl^−^ > Br^−^ > I^−^
ClC2	Plasma membrane	Leukodystrophy	2–3	Cl^−^ > Br^−^ > I^−^ > Cl^−^ (in cell swelling)
ClC3	Plasma membrane and late endosomes	Degeneration of CNS and retina	∼40	Cl^−^ > I^−^
ClC4	Endosomes	Epilepsy	∼1	Cl^−^ > I^−^
ClC5	Endosomes	Dent’s disease and impaired renal endocytosis		NO_3_^−^ > Cl^−^ > Br^−^ > I^−^
ClC6	Late endosomes	Lysosomal storage in neurons	∼100 (from bilayer recordings)	–
ClC7	Lysosomes	Osteopetrosis, CNS, and retina degeneration		–
ClCKa	Plasma membrane of inner ear and kidney	Diabetes insipidus	–	Cl^−^ > Br^−^ > NO_3_ > I^−^
ClCKb	Plasma membrane of inner ear and kidney	Bartter’s syndrome	20–25 (with barttin subunit)	Br^−^ > I^−^ > Cl^−^
CFTR	Plasma membrane	Cystic fibrosis, acute pancreatitis, chronic obstructive pulmonary disease, and the hyper-responsiveness in asthma	∼10	Br^−^ ≥ Cl^−^ > I^−^ > F^−^
GABAARs	Plasma membrane	Neurological functions, seizures, hypotonia, and hyperreflexia	∼28, 18, and 12	Cl^−^ > HCO_3_^−^
ORCC	Plasma membrane	Cystic fibrosis	30–60	Cl^−^ ≥ Br^−^ > I^−^
TMEM16A; Anoctamin-1; ANO1	Plasma membrane	Up-regulation in gastrointestinal stromal tumors (GISTs), in breast cancer, and in head and neck squamous cell carcinomas (HNSCCs); up-regulated in asthma	1–14	I^−^ > NO_3_^−^ > Br^−^ > Cl^−^ > F^−^ > CH_3_SO_4_
TMEM16B; Anoctamin-2; ANO2	Plasma membrane	Anxiety modulation	∼10	SCN^−^ (14) > I^−^ > NO_3_^−^ > Br^−^
TMEM16F; Anoctamin-6; ANO6	Plasma membrane	Mutated in Scott syndrome	1–3	I^−^ > Br^−^ > Cl^−^ > F^−^ > aspartate
CLIC1	Cytoplasm, exosomes, plasma membrane, intracellular membrane, mitochondria, and nucleoplasm	Myelodysplastic syndrome and several cancers	35–50 (from bilayer recordings) with sub states	I^−^ > SCN^−^ ≥ Cl^−^ ≥ NO_2_^−^ and NO_3_^−^≥ Br^−^ ≥ F^−^ (in symmetrical ionic conditions) I^−^ > F^−^ = SCN^−^ > Cl^−^ = NO_2_^−^ and NO_3_^−^ = Br^−^ (in asymmetrical ionic conditions)
CLIC2	Cytoplasm, nucleus, and endoplasmic reticulum	X-linked cognitive disability, congestive heart failure, cardiomegaly, erythematosus, seizures, myopia, and atrial fibrillation	30–40 (from bilayer recordings)	Cl > Choline
CLIC3	Nucleus, exosome, and plasma membrane	Fetal growth restriction, pre-eclampsia, and breast cancer	∼1–2 nS	–
CLIC4	Cytoplasm, mitochondrial associated membrane (cardiomyocytes), nucleus, exosomes, golgi apparatus, plasma membrane, and intracellular membrane	Several cancers, benign familial infantile seizures, and pulmonary hypertension	10, 30, and 57 (from bilayer and tip dip recordings)	–
CLIC5	Nucleus, inner mitochondrial membrane (cardiomyocytes), exosomes, Golgi apparatus, plasma membrane, intracellular membrane, and secretory vesicles in renal glomeruli	Renal dysfunction, juvenile myoclonic epilepsy, migraine, macular degeneration, and childhood acute lymphoblastic leukemia	∼105 (from bilayer recordings)	–
CLIC6	Cytoplasm, exosomes, nucleus, and plasma membrane	Familial goiter and developmental delay	1–3	Cl^−^ > Br^−^ > F^−^
VDAC1	Plasma membrane and mitochondrial outer membrane	Cystic fibrosis, mitochondrial myopathy, and calcium-induced neurotoxicity	200–250	Cl^−^ > K^+^ > Na^+^ > glutamate > ATP > acetylcholine > dopamine
VDAC2	Mitochondrial outer membrane	Alzheimer’s, thyroid cancer, temporal lobe epilepsy (TLE), hypoxia, iron deprivation, and adipogenesis	1–2 nS	Cl^−^ > K^+^ (from nanodiscs)
VDAC3	Mitochondrial outer membrane	Hepatocellular carcinoma	3–4 nS	Cl^−^ > K^+^ (from nanodiscs)
IMAC	Mitochondrial inner membrane	Type 2 diabetes, Parkinson’s disease, atherosclerotic heart disease, stroke, Alzheimer’s disease, and cancer	107–150	Cl^−^ > SO_4_^2−^> P_i_ ≅ 1,2,3-BTC > 1,3,5-BTC
VRAC; VSOR; VSOAC	Plasma membrane	Angiogenesis, cancer, ischemic, and apoptosis	10–90	I^−^ ≥ Br^−^ > Cl^−^ > F^−^ > taurine > glutamate
PAC; ASOR; PAORAC; TMEM206	Endosomes	Ischemic stroke, cancer, and hypoxia	40–10	SCN^−^ > I^−^ > NO_3_^−^ > Br^−^ > Cl^−^

Numerous chloride channels and transporters are highlighted by their localization in the cell, pathophysiology, conductance, and permeability. ClC1, chloride channel 1; ClC2, chloride channel 2; ClC3, chloride channel 3; ClC4, chloride channel 4; ClC5, chloride channel 5; ClC6, chloride channel 6; ClC7, chloride channel 7; CLIC1, chloride intracellular channel 1; CLIC2, chloride intracellular channel 2; CLIC3, chloride intracellular channel 3; CLIC4, chloride intracellular channel 4; CLIC5, chloride intracellular channel 5; CLIC6, chloride intracellular channel 6; ClCKA, kidney-specific chloride channel A; ClCKB, kidney-specific chloride channel B; CFTR, cystic fibrosis transmembrane conductance regulator; GABAARs, γ-aminobutyric acid type A receptors; IMAC, mitochondrial inner membrane anion channel; ORCC, outward rectifying Cl^−^ channel; PAC, proton-activated Cl^−^ channel; PAORAC/ASOR, acid-sensitive outwardly-rectifying anion channel; TMEM16A/ANO1, calcium-activated chloride channel ANO1/TMEM16A; TMEM16B/ANO2, calcium-activated chloride channel ANO2/TMEM16B; TMEM16F/ANO6, calcium-activated chloride channel ANO6/TMEM16F; VDAC1, voltage-dependent anion-selective channel 1; VDAC2, voltage-dependent anion-selective channel 2; VDAC3, voltage-dependent anion-selective channel 3; VRAC, volume-regulated anion channel; VSOR, volume-sensitive outwardly rectifying anion; VSOAC, volume-sensitive organic osmolyte/anion channel [4,67,68,84,98,103,122,123,126,135,136,139,140,144,145,165,168,176–194].

In the brain, Cl^−^ was characterized for regulating the circadian rhythm [78]. Circadian rhythm is regulated by the suprachiasmatic nucleus (SCN), which predominantly comprises of GABAergic neurons. In SCN, GABAergic neurons elicit excitatory responses, which are facilitated by an increase in intracellular Cl^−^ levels [79]. Also, the Cl^−^ levels in cortical pyramidal neurons were found to be associated with the sleep–wake cycle [78]. During the sleep part of the cycle, Cl^−^ levels decrease, but during the wake part of the cycle, the levels increase [78]. The increase in Cl^−^ levels during wakefulness is associated with inhibitory synaptic transmission in the cortex [80]. In sleep-deprived animals, alterations in Cl^−^ levels were found to be sufficient to correct the drop in their cognitive performance levels [80]. The major mechanism in this Cl^−^mediated sleep–wake regulation is the equilibrium potential for the GABA_A_ receptor [80,81]. Decreasing Cl^−^ to hyperpolarizing equilibrium potential for the GABA_A_R in animals deprived of sleep was sufficient to restore performance levels [80]. These findings indicate that targeting Cl^−^ regulatory mechanisms could improve therapeutic effects in sleep disorders.

### Chloride in the liver

In the liver, there is limited information available on the physiological role of Cl^−^. Cl^−^ levels in newborns were found to be 55 mM, whereas in adults they were reported to be at 38.3 mM (Figure 1) [82,83]. Surprisingly, in the same tissue, although the cytosolic Cl^−^ levels were found to be higher, these levels still showed a general decrease from 60 mM in newborns to 38 mM in adults [83]. The alteration in levels of Cl^−^ could be attributed to the food or ion intake or to different expressions of ion channels and transporters in adults as compared with newborns. Additionally, mitochondria in the liver cells of newborns had approximately 5 mM of Cl^−^, approximately 30-fold lower than the cytosolic Cl^−^ levels [83]. However, with age, the Cl^−^ levels do not show as strong of an inverse trend in the mitochondria as observed for cytosolic Cl^−^ levels [83]. Though there is a strong electrochemical gradient between the cytosol and mitochondria for Cl^−^, the levels indicate a tight regulation, possibly by ion channels and transporters [83].

Hepatocytes have Cl^−^ channels in several intracellular compartments as well as at the plasma membrane [84]. The regulation of intracellular organelle acidification and cell volume depends on these channels [84]. Ca^2+^-activated Cl^−^ channels have been found in the plasma membranes of hepatocytes [84]. The mitochondrial voltage-dependent anion channel, members of the newly discovered CLIC family of intracellular chloride channels (CLIC-1 and CLIC-4), members of the ClC channel family (ClC-2, ClC-3, ClC-5, and ClC-7), and a newly discovered intracellular channel, MCLC (Mid-1 related chloride channel), are among the Cl^−^ channel molecules that have been demonstrated to be expressed in hepatocytes [11,83,85,86].

There has not been much research done on the significance of Cl^−^ alterations for the prognosis of cirrhosis patients (Figure 1). In critically ill patients with decompensated cirrhosis, two independent studies found hypochloremia to be associated with short-term mortality, but not hyponatremia [87,88]. Interestingly, hypochloremia was found to be a more significant indicator of a patient’s prognosis than hyponatremia [89].

### Chloride in the lungs

Cl^−^ levels in the lungs are essential to maintaining membrane excitability, transepithelial transport, and homeostasis of ions as well as water [72]. The Cl^−^ concentration in lung cells is maintained by a plethora of ion channels and transporters [90]. The earliest diagnosis involving Cl^−^ was made for cystic fibrosis transmembrane conductance regulator (CFTR), a condition where the sweat of affected children tastes saltier than normal children [91]. In CFTR patients, there is a notable increase in Cl^−^ levels of sweat to 60 mM as opposed to normal levels of 30 mM (Figure 1) [91]. If the Cl^−^ is not moving in the correct direction, water is unable to hydrate the surface of cells. This causes thick and sticky mucus to cover the cells, resulting in many of the symptoms related to cystic fibrosis. In addition, patients with a chronic cough have been reported to have both reduced pH and Cl^−^ levels [92].

In the lungs, Cl^−^ and water move paracellularly to maintain both electroneutrality and osmotic balance [93]. Passive absorption of Cl^−^ by various pathways is driven in response to the electrical driving force generated by active Na^+^ absorption. However, transepithelial Cl^−^ transporters are implicated in active alveolar secretion and cardiogenic edema formation [93]. In airways surface the liquid Cl^−^ concentration is approximately 123 mM [94], and in the airway epithelia, the range is from 30 to 50 mM [95]. Furthermore, it was shown that both transepithelial alveolar Cl^−^ and fluid flux can reverse from an absorptive to a secretory mode in lung hydrostatic stress [93]. When Cl^−^ was replaced with iso-osmolar NO_3_^−^, it attenuated alveolar fluid clearance [93]. Cl^−^ must follow electroneutrality in lung cells [93]. Failure to maintain electroneutrality limits transepithelial Na^+^ flux, hence, affecting alveolar fluid clearance [93]. The idea of a significant role for transepithelial Cl^−^ transport in alveolar fluid secretion is further supported by the fact that alveolar fluid secretion is prevented in Cl^−^ free perfused lungs [93].

Cl^−^ channels are highly expressed in the lung in both the lung parenchyma and the pulmonary blood vessels. They can develop pulmonary diseases (Figure 1) because of their compromised function or regulation [90]. The major challenges in the identification of Cl^−^ channels and transporters are weak, non-selective inhibitors or a lack of genetic studies [9]. The major channels and transporters implicated in lung cells are TMEMs [96], cAMP-activated Cl^−^channels [97], ClC family [98], ligand-gated Cl^−^ channels [99], SLC26 [100], CLIC4 [101,102], and CLIC6 [103].

### Chloride in the muscles

Cl^−^ regulates the excitability of muscle cells in skeletal muscles via their movements in and out of cells [32,104–107]. The electrical potential of the cells is stabilized by this flux, preventing abnormal muscle contraction. Although the resting Cl^−^ conductance is not high, Cl^−^ levels increase the excitability of cardiac cells in cardiac muscle, also known as the myocardium [108] (Figure 1).

Various vascular responses involve Cl^−^ currents, indicating the existence Cl^−^ channels such as transmembrane protein 16 (TMEM16)/anoctamin (ANO), bestrophins, voltage-gated Cl^−^ channels (CLCs), cystic fibrosis (CF) transmembrane conductance regulator (CFTR) [109–112]. Vascular smooth muscle cells have been found to harbor all known Cl^−^ channel families, with the exception of the GABA-/glycine-receptor family [109]. It has been proposed that at least one member of the voltage-activated ClC family, ClC-3, is involved in cell proliferation, myogenic constriction, and anti-apoptotic activity in rat vascular smooth muscle cells (VSMCs) [113]. VSMCs also exhibit the transmembrane conductance regulation associated with cystic fibrosis [114].

Myotonia congenita (MC), a genetic neuromuscular channelopathy, affects the skeletal muscle fibers, which are the striated muscles under the control of the somatic nervous system [115]. It is also associated with the abnormal functioning of Cl^−^ channels such as ClC-1 (Figure 1) [32,116,117]. Myotonia, the disease’s hallmark, is defined as a delay or failure of relaxation in contracted skeletal muscle [115]. It causes prolonged rigidity, leading to cramping, stiffness, and muscle hypertrophy [115]. The CLCN1 gene, which codes for voltage-gated chloride (CIC-1) channels in the sarcolemmal membrane, is mutated in MC [116]. Repetitive depolarization and myotonia are caused by abnormal hyperexcitability of skeletal muscle cells due to defective CIC-1 channels [118].

In addition to VSMCs, Cl^−^ channels have also been discovered in cardiac tissues. Levels of Cl^−^ in the serum can determine the survival outcome after cardiac insults such as a heart attack or chronic heart failure [18–21,23]. Pharmacological and genetic approaches have indicated that IAA-94-sensitive Cl^−^ channels such as chloride intracellular channels (CLICs), CLIC1, CLIC2, CLIC4, and CLIC5 are present in the cardiac tissue [119–123]. Blocking or absence of these channels increased myocardial infarction after ischemia and reperfusion injury [119,124–127]. Similarly, voltage-dependent anion channel (VDAC) ablation also results in dilated cardiomyopathy and cell death [128–130]. In skeletal muscle fibers, intracellular Cl^−^ levels have a small potentiating effect on the Ca^2+^ release, which influences the cellular Ca^2+^ levels [131]. Pharmacological approaches have also implicated Cl^−^ fluxes in charge compensation in smooth muscle cells [132]. It was further shown that different channels and transporters are involved in smooth and cardiac muscle cells [132].

### Chloride in intracellular organelles

Cl^−^ levels in the ECF are 110 mM, but in the cytosol, the levels are as low as 45 mM [133]. With the advent of new nano sensors and technologies, it is possible to quantify the absolute concentration of Cl^−^ concentrations in various cellular compartments [133,134]. The Cl^−^ concentration inside cellular organelles is tightly regulated for their physiological function [65]. The regulation is vital for maintaining ionic homeostasis and water concentrations. Different Cl^−^ concentrations in different cellular compartments are provided in Figure 2.

### Chloride ion channels and transporters

Cl^−^ is moved across the cellular membrane through ion channels and transporters [135]. They are activated by pH, Ca^2+^, voltage, and volume [4]. After being ignored for several decades, Cl^−^ channels and transporters have been discovered through the cloning of VDACs [136–138], ClC family [139,140], GABA_A_ receptors [141–143], and CLIC proteins [144–146], as well as through the identification of mutations in the cystic fibrosis transmembrane conductance regulator (CFTR) [147]. So far, over 53 Cl^−^ transporting proteins have been identified [90]. These ion channels and transporters are associated with several human disorders or disease-like symptoms (Table 2) [148]. The major challenge in the Cl^−^ channel and transport fields is the lack of pharmacological agents that can activate, block, inhibit, or facilitate membrane trafficking of these proteins. A major multidisciplinary effort is required to push for Cl^−^ channels and transporters as drug candidates. Most of the Cl^−^ channels and transporters are listed as potential drug targets that are not extensively studied [149]. Recently, a few Cl^−^ transporters have been identified as targets of FDA-approved drugs [9,11]. For example, diuretics target SLC12 cation-Cl^−^ co-transporters, which are used to reduce volume overload in hypertension and heart failure [150]. Barbiturates and benzodiazepines are known to target GABA-gated Cl^−^ channels, and are commonly used for anxiety disorders, depression, and insomnia [151]. Ivacaftor was approved in 2012, and several correctors approved in 2015 for CFTR were highly specific steps to exclusively target Cl^−^ channels [152]. More importantly, several drug candidates, such as acamprosate, alprazolam, bendroflumethiazide, benthiazide, bumetanide, butabarbital, butalbital, chlorothiazide, chlordiazepoxide, chlorthalidone, clobazam, clonazepam, clorazepic acid, crofelemer, cyclothiazide, desflurane, diazepam, enflurane, estazolam, eszopiclone, ethacrynic acid, ethchlorvynol, etomidate, flumazenil, flurazepam, furosemide, glutethimide, halazepam, halothane, hydrochlorothiazide, hydroflumethaiazide, indapamide, isoflurane, ivermectin, lindane, lorazepam, lubiprostone, lumacaftor, meprobamate, metharbital, methohexital, methoxyflurane, methyclothiazide, methyprylon, metolazone, midazolam, oxazepam, pentobarbital, polythiazide, prazepam, primidone, propofol, quazepam, quinethazone, secobarbital, sevoflurane, talbutal, temazepam, thiamylal, thiopental, tiagabine, topiramate, torsemide, triazolam, trichloromethiazide, triclofos, targeting Cl^−^ channels, and transporters are listed with FDA clinical trial efforts [153]. There are several Cl^−^ channels and transporters characterized as summarized in Table 2.

## Perspectives

1. Cl^−^ are major anions in the body, and recent literature suggests that a decrease in Cl^−^ levels in the body can result in detrimental effects [6,9,18,22,50,65,67,76,90,91,115,120,154,155]. A specific mechanism to increase Cl^−^ in organs could improve the survival rate and the health of human beings.
2. Cl^−^ levels vary in different organ systems during development; however, there is no clear information on how these chloride ions are important in development and aging [3,4,9,23,75,94,107,112,120,133,156–161]. Recognition of variability in ion concentration during development and aging will facilitate novel targets for development-related pathological conditions.
3. Cl^−^ levels in organelles and cells are tightly regulated by ion channels and transporters. Identification and regulatory mechanisms of these channels and transporters hold the key to modulating cellular and extra-cellular Cl^−^ levels [4,71,72,83,133,145,146,162–175].

## Data Availability

Not applicable

## Abbreviations

ANO: anoctamin
CF: cystic fibrosis
CFTR: CF transmembrane conductance regulator
CLC: Cl^−^ channel
ECF: extracellular fluid
NHE: Sodium Hydrogen antiporter 3
PCT: proximal convoluted tubule
TMEM16: transmembrane protein 16
VDAC: voltage-dependent anion channel
VSMC: vascular smooth muscle cell
